# Flexible Lead-Free Ba_0.5_Sr_0.5_TiO_3_/0.4BiFeO_3_-0.6SrTiO_3_ Dielectric Film Capacitor with High Energy Storage Performance

**DOI:** 10.3390/nano11113065

**Published:** 2021-11-14

**Authors:** Wenwen Wang, Jin Qian, Chaohui Geng, Mengjia Fan, Changhong Yang, Lingchao Lu, Zhenxiang Cheng

**Affiliations:** 1 Shandong Provincial Key Laboratory of Preparation and Measurement of Building Materials, University of Jinan, Jinan 250022, China; wangwenwen_0717@163.com (W.W.); j28_qian@163.com (J.Q.); g1902803618@163.com (C.G.); fan15176103198@163.com (M.F.); mse_lulc@ujn.edu.cn (L.L.); 2 Institute for Superconducting and Electronic Materials, Australian Institute for Innovative Materials, University of Wollongong, Innovation Campus, North Wollongong, NSW 2500, Australia; cheng@uow.edu.au

**Keywords:** flexible, film capacitor, Ba_0.5_Sr_0.5_TiO_3_/0.4BiFeO_3_-0.6SrTiO_3_, energy storage properties

## Abstract

Ferroelectric thin film capacitors have triggered great interest in pulsed power systems because of their high-power density and ultrafast charge–discharge speed, but less attention has been paid to the realization of flexible capacitors for wearable electronics and power systems. In this work, a flexible Ba_0.5_Sr_0.5_TiO_3_/0.4BiFeO_3_-0.6SrTiO_3_ thin film capacitor is synthesized on mica substrate. It possesses an energy storage density of *W*_rec_ ~ 62 J cm^−3^, combined with an efficiency of *η* ~ 74% due to the moderate breakdown strength (3000 kV cm^−1^) and the strong relaxor behavior. The energy storage performances for the film capacitor are also very stable over a broad temperature range (−50–200 °C) and frequency range (500 Hz–20 kHz). Moreover, the *W*_rec_ and *η* are stabilized after 10^8^ fatigue cycles. Additionally, the superior energy storage capability can be well maintained under a small bending radius (*r* = 2 mm), or after 10^4^ mechanical bending cycles. These results reveal that the Ba_0.5_Sr_0.5_TiO_3_/0.4BiFeO_3_-0.6SrTiO_3_ film capacitors in this work have great potential for use in flexible microenergy storage systems.

## 1. Introduction

At present, the energy crisis and environmental pollution have aroused widespread concern. In order to solve these problems, it is necessary to develop and utilize clean and sustainable energy sources and energy storage devices [1,2,3,4]. At present, advanced energy storage techniques include batteries, superconducting magnetic energy storage systems and electrochemical/dielectric capacitors [5,6]. Among them, dielectric capacitors are attracting immense research interest in pulsed power systems due to their unique features of high-power density (up to 10^8^ W kg^−1^) and short charge−discharge time (10^−3^–10^−6^ s) [7,8,9,10,11,12].

For dielectric capacitors, the energy storage capability (recoverable energy storage density *W*_rec_, energy storage efficiency *η*) can be calculated by [13,14]:

(1) $$W_{\mathrm{rec}}=\int_{P_{r}}^{P_{m}}E\;dP$$

(2) $$W=\int_{0}^{P_{m}}E\;dP$$

(3) $$\eta =\frac{W_{\mathrm{rec}}}{W}\times 100\%=\frac{W_{\mathrm{rec}}}{W_{\mathrm{rec}}+W_{\mathrm{loss}}}\times 100\%$$

where *W*, *W*_loss_, *E*, *P*_m_ and *P*_r_ represent the total energy storage density, the energy loss density, applied electric field, the maximum polarization and the remanent polarization during the discharge process, respectively. Therefore, *W*_rec_ can be improved by increasing the difference between *P*_m_ and *P*_r_ and enhancing the electric breakdown strength (*E*_b_).

Currently, commercial biaxially oriented polypropylene (BOPP) has been widely employed in power inverter capacitor systems. The bottleneck problems faced by BOPP are its limited *W*_rec_ of (<2 J cm^−3^) and poor thermal stability (<80 °C), which inevitably burden the weight of the device, increase the difficulty of structure design and narrow the working temperature window [15,16,17]. In recent years, inorganic dielectric capacitors using oxide thin films as functional elements have been widely studied due to their relatively high *W*_rec_ compared with organic dielectrics. Recent investigations into the energy storage characteristics of several representative dielectric capacitors have been summarized and listed in Table 1. Obviously, energy storage properties such as *W*_rec_ and *η* have been studied in film capacitors containing BiFeO_3_(BFO)/BaTiO_3_(BTO)/SrTiO_3_(STO). For example, Huang et al. reported that introducing Sr in BTO can effectively reduce the coercive field (*E*_c_) and *P*_r_, leading to an enhanced *W*_rec_ and *η* [18]. Pan et al. demonstrated that a giant *W*_rec_ of ~70 J cm^−3^, together with a high *η*, can be achieved in lead-free 0.4BFO-0.6STO films through domain engineering [6]. They also designed a 0.25BFO-0.3BTO-0.45STO ternary solid solution system, in which a high *W*_rec_ of up to 112 J cm^−3^ and an *η* of ~80% were obtained coexistence of the rhombohedral and tetragonal nanodomains in a cubic matrix [19]. Moreover, in Bi doped STO, ferroelectric relaxation behavior is observed, which plays a decisive role in the high energy storage property, especially the ultra-high *η* [20,21,22,23].

With the rapid development of electronic devices leaning toward miniaturization and integration, flexible electronics have been an active research topic in various areas due to their distinctive advantages of being portable, lightweight, foldable, stretchable and even wearable [39,40,41,42,43,44]. Flexible and microscale dielectric capacitors as energy storage components are indispensable especially in next-generation micro-electrical power systems. Nevertheless, most inorganic dielectric films are grown on rigid substrates due to the lack of suitable flexible substrates. Common flexible polymer substrates, such as polyimide (PI) or polyethylene naphthalate (PEN), have very excellent mechanical compliance but they cannot withstand the high crystallization temperature of inorganic films due to their low melting point (PI ~ 520 ℃, PEN ~ 270 ℃). Fortunately, the emergence of MICAtronics provides a new idea to realize flexibility in oxide functional films with two-dimensional mica as the substrate. This is due to the fact mica possesses ultrahigh melting point (1000 ℃–1100 ℃) and atomically flat surface, making it more compatible with the inorganic thin film preparation process.[45,46,47]. However, a flexible dielectric film capacitor consisting of BFO, BTO, and STO elements has rarely been reported.

Considering that 0.4BiFeO_3_-0.6SrTiO_3_ (0.4BFO-0.6STO) is a relaxor ferroelectric with an attractive relaxor feature and Ba_0.5_Sr_0.5_TiO_3_ (BST) is paraelectric with low dielectric loss and high breakdown strength [6,48,49], a multilayer structure of BST/0.4BFO-0.6STO is envisaged in this work based on the two potential energy storage elements. A series of systematic studies about the energy storage capability are undertaken on the designed film, which is deposited on flexible mica substrate using a sol-gel method. The capacitor shows a high *W*_rec_ of ~62 J cm^−3^ and an *η* of ~74% simultaneously due to its relatively high *E*_b_ of 3000 kV cm^−1^ and strong relaxor behavior. Satisfyingly, prominent mechanical-bending resistance is also realized in the flexible BST/0.4BFO-0.6STO film, in which the *W*_rec_ and *η* have no obvious deterioration under various bending radii (*r* = 12–2 mm) and even after 10^4^ bending cycles at *r* = 4 mm.

## 2. Materials and Methods

### 2.1. Film Fabrication

Firstly, the flexible mica substrate coated with bottom electrode was provided for depositing dielectric thin film. The fluorophlogopite mica [KMg_3_(AlSi_3_O_10_)F_2_] was purchased from Changchun Taiyuan Fluorophlogopite Co., Ltd. (Changchun, China). The mica sheet was washed with ethanol and water to get a cleaned surface. Then, a 20 nm thick Pt layer was sputtered onto the surface under a 30 mA current in Ar atmosphere of 0.05 mbar, to be used as the bottom electrode.

The multilayer BST/0.4BFO-0.6STO thin film was fabricated on Pt/mica substrate by sol-gel. Precursor solutions of BFO, BTO and STO were prepared, respectively, with the use of bismuth nitrate pentahydrate, iron nitrate nonahydrate, strontium acetate, barium acetate and tetrabutyl titanate. Ethylene glycol and acetic acid were selected as solvents to dissolve the solid raw materials. Here, 5 mol% excess bismuth was added to compensate for element volatilization during the high temperature treatment. Subsequently, the tetrabutyl titanate and acetylacetone were added into the solution. Meanwhile, 2 mol% manganese acetate tetrahydrate was added to each solution to improve the electrical resistivity of the film. The final concentration of each precursor solution was 0.15 M. Then, we used proportionable BFO and BTO solutions, separately mixed with STO, to form solutions of 0.4BFO-0.6STO and BST. The solutions were stirred with a magnetic stirrer for 12 h and further aged for another 48 h. The BST layer was first spin-coated on the substrate, and 0.4BFO-0.6STO layers were then deposited in situ on top of the BST layer. Both layers were dried at 200 °C for 2 min, successively. Subsequently, each layer was pyrolyzed on a hot plate at 300 °C for 5 min and annealed in a mini tubular furnace at 700 °C for 10 min. Both components (0.4BFO-0.6STO and BST) were spin coated alternately 10 times, with the ultimate sample consisting of twenty dielectric layers. For electrical measurements, Au top electrodes with a diameter of about 200 μm were sputtered through a shadow mask to form the capacitor structure. Finally, a simple mechanical peeling process was conducted to realize flexibility in the film by tearing off the bottom mica layer to reduce the thickness to ~10 μm.

### 2.2. Characterization

The crystalline structure of BST/0.4BFO-0.6STO film was monitored in the 2*θ* range of 20–60° by an X-ray diffractometer (XRD, D8 ADVANCE, Karlsruhe, Germany). During the XRD test, the scanning rate was 0.12 s per step, and the number of scanning steps was a total of 2054 steps. The surface morphology was studied by a tapping mode atomic force microscope (AFM, Bruker Dimension Icon, Santa Barbara, CA, US). The cross-sectional microstructure and EDS spectrum were studied by a field-emission scanning electron microscope (FESEM, ZEISS Gemini300, Oberkochen, Germany) using 2 kV acceleration voltage and 10 kV acceleration voltage, respectively. The polarization electric field (*P-E*) relations were examined by a standard ferroelectric tester (aixACCT TF3000, Aachen, Germany) at room temperature, at a frequency of 10 kHz. In regard to temperature-dependence polarization properties, the loops were measured from –50 to 200 °C with a temperature interval of 25 °C at 10 kHz. The frequency dependent *P-E* measurements were conducted at room temperature from 500 Hz to 20 kHz. The dielectric properties were characterized by way of an impedance analyzer (HP4294A, Agilent, Palo Alto, CA, USA) at a temperature range of −50 to 250 °C from 1 kHz to 100 kHz, with an oscillation voltage of 1.0 V. The temperature-related electrical measurements were carried out with the assistance of a temperature-controlled probe station (Linkam-HFS600E-PB2, London, UK) with a heating rate of 8 °C min^−1^. The cyclic bending tests were realized by using a homebuilt stepper motor control system. The fast energy discharge behavior was evaluated by using a home-built resistance-capacitance (RC) circuit with a load resistance of 100 kΩ.

## 3. Results

**Figure 1:**
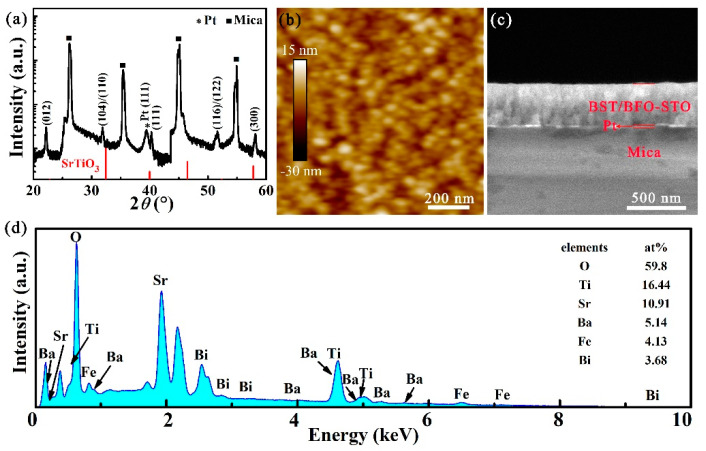
(**a**) X-ray diffraction pattern in the 2*θ* range of 20–60°, (**b**) AFM, (**c**) Cross-sectional SEM images and (**d**) EDS spectrum of BST/0.4BFO-0.6STO thin film.

Figure 1a shows the XRD pattern of BST/0.4BFO-0.6STO film grown on Pt/mica substrate. Visually, the film possesses a single perovskite phase with no detectable secondary phase, suggesting that the film can be well crystallized. Figure 1b shows the surface AFM image of BST/0.4BFO-0.6STO film. The average surface roughness (*R*_a_) and root mean square roughness (*R*_rms_) of the film are determined to be 2.54 nm and 2.06 nm, respectively, which may be attributed to the atomic flatness of mica substrate and high crystallinity of the film. The obtained roughness is at the same level of the reported inorganic films [36,47]. The grain size distribution of the film is analyzed using the Nano Measurer software by randomly selecting 100 grains. In addition, the average grain size value estimated from the AFM image is 53.26 nm. Figure 1c shows the cross-sectional image of multilayer film. From it, the film’s thickness can be determined to be ~350 nm. Furthermore, the thickness of the bottom Pt electrode is about 20 nm. The ultimate film composition of the BST/0.4BFO-0.6STO film is determined via EDS spectrum, as displayed in Figure 1d. The atomic percentages (atom%) of O, Ti, Sr, Ba, Fe and Bi are 59.80, 16.44, 10.91, 5.14, 4.13 and 3.68, respectively, confirming a near perfect BST/0.4BFO-0.6STO stoichiometry.

**Figure 2:**
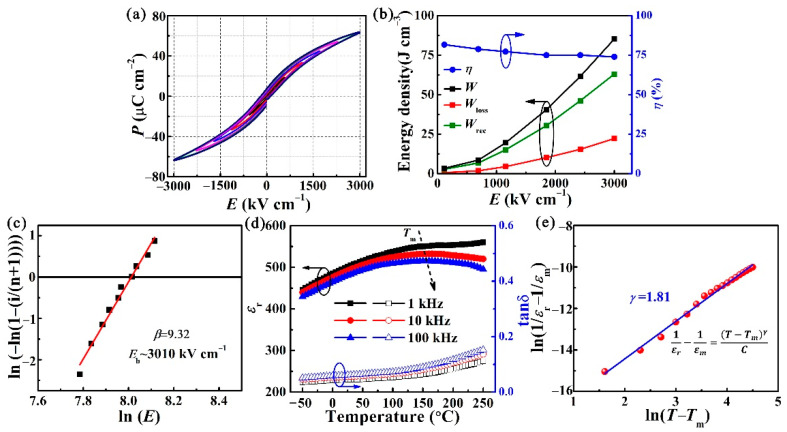
(**a**) The *P-E* loops for BST/0.4BFO-0.6STO under various applied electric fields. (**b**) The calculated *W*, *W*_rec_, *W*_loss_ and *ƞ* values as functions of the electric field. (**c**) Two-parameter Weibull analysis of dielectric breakdown strength. (**d**) Temperature-dependent *ε*_r_ and tan*δ* under the frequency range of 1 kHz–100 kHz and the temperature range from −50 to 250 °C. (**e**) ln(1/*ε*_r_−1/*ε*_m_) as a function of ln(*T*−*T*_m_).

The bipolar *P-E* loops for the BST/0.4BFO-0.6STO film in Figure 2a are measured from a low electric field to 3000 kV cm^−1^ at room temperature, at a frequency of 10 kHz. Figure 2b presents the corresponding energy storage parameters of the *W*, *W*_rec_, *W*_loss_ and *η* at various electric fields determined by *P-E* loops. The *W*_rec_ and *η* extrapolated from a bipolar *P*-*E* loop under *E*_b_ (3000 kV/cm) are 62 J cm^−3^ and ~74%, respectively, which is a relatively high level among the flexible dielectric films [37,50]. It can be seen that the *P*_m_ and *P*_r_ are 63.52 μC cm^−2^ and 6.73 μC cm^−2^, respectively, which contributed a great Δ*P* = 56.79 μC cm^−2^, and the result is beneficial for energy storage performance. This small *P*_r_ can be due to the fact that BiFeO_3_-SrTiO_3_ is a relaxor ferroelectric and BST is paraelectric.

The Weibull distribution of *E*_b_ can be obtained through the following formula:

(4) $$X_{i}=Ln\left(E_{i}\right)$$

(5) $$Y_{i}=Ln\left(-Ln\left(1-\frac{i}{n+1}\right)\right)$$

where *E_i_*, *i* and n signify the breakdown electric field, the serial number of tested specimens and the total number of tested specimens, respectively. Based on the Weibull distribution function, there exists a linear relationship between X_i_ and Y_i_. The mean *E*_b_ for thin film can be extracted from the intersect points of the fitting lines and the horizontal axis at *Y*_i_ = 0. The solid fitting straight line shown in Figure 2c is the Weibull analysis result of ten data gathered from our thin film. It can be observed that the slope parameter *β* is 9.32, which indicates both the good composition uniformity and high dielectric reliability of BST/0.4BFO-0.6STO [47]. The average *E*_b_ extracted by the horizontal intercept is about 3010 kV cm^−1^. The temperature-dependent dielectric permittivity (*ε*_r_) and loss (tan *δ*) of the BST/0.4BFO-0.6STO film exhibit nearly flat permittivity peaks and frequency dispersion over the range of −50 to 250 °C, as shown in Figure 2d, indicating the relaxor characteristic. Notably, a broad and smeared peak of maximum *ε*_r_ appears, especially near 150 °C. With increasing frequency, the maximum dielectric permittivity (*ε*_m_) at *T*_m_ decreases and *T*_m_ shifts to a higher temperature, which are important signatures of relaxor behavior [48]. To evaluate the relaxor dispersion degree, a modified Curie–Weiss equation of 1/*ε*_r_ −1/*ε*_m_ = (*T* − *T*_m_)^γ^/C can be used to estimate the relaxor dispersion degree, where *ε*_m_ represents the maximum dielectric constant at *T*_m_, C is the Curie constant and *γ* is the relaxor diffuseness factor. Generally, *γ* = 1 represents a normal ferroelectric, 1 ≤ *γ* ≤ 2 represents the relaxor ferroelectric behavior and *γ* = 2 is valid for a classical ferroelectric relaxor [49]. After calculation, the *γ* for the film is 1.81 in Figure 2e, further evidencing the relaxor feature.

**Figure 3:**
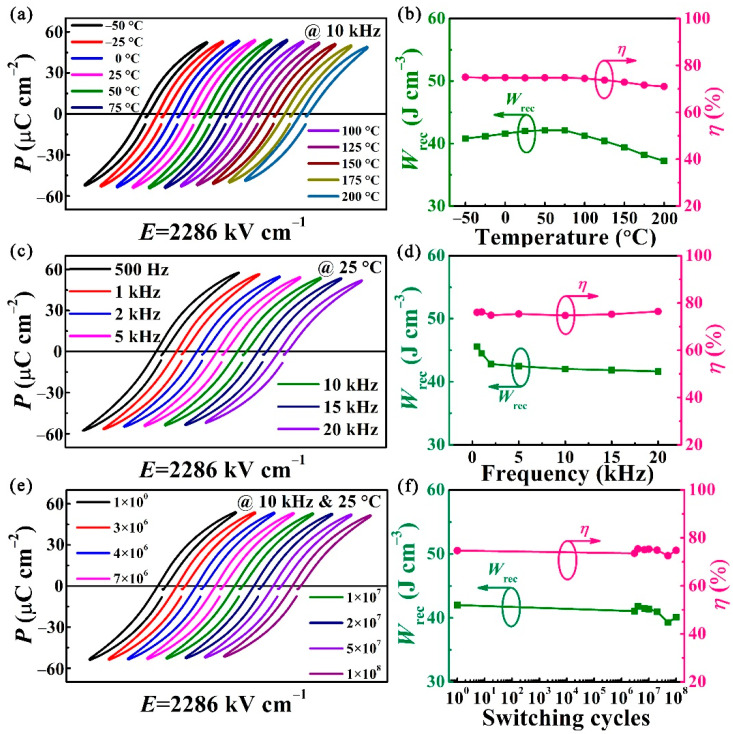
(**a**) The *P-E* curves and (**b**) the corresponding *W*_rec_ and *ƞ* measured from −50 to 200 °C at 2286 kV cm^−1^. (**c**) The *P-E* curves and (**d**) the corresponding *W*_rec_ and *ƞ* with various frequencies measured under 2286 kV cm^−1^. (**e**) The *P-E* curves and (**f**) the corresponding *W*_rec_ and *ƞ* during the 10^8^ fatigue cycles at 2286 kV cm^−1^. The measurements are realized at about 76% of *E*_b_.

The temperature and frequency stability, as well as the antifatigue property for the sample, are evaluated, as shown in Figure 3. Firstly, the *P-E* hysteresis loops are measured at 10 kHz under 2286 kV cm^−1^ in the temperature range of −50 to 200 °C. As illustrated in Figure 3a, the *P-E* loops almost preserve their pinched shape, and the *P*_m_ and *P*_r_ values have tiny changes. Correspondingly, the *W*_rec_ and *η* of BST/0.4BFO-0.6STO films fluctuate slightly by 11% and 5% as shown in Figure 3b, which indicates the excellent thermal stability of the energy performance of the film. In practical application, it is necessary to meet the working temperature range of capacitors; for example, when in use in the fields of hybrid electric vehicles (~140 °C), drilling operations (150–200 °C), or in outer space and high-altitude aircraft (~−50 °C) [1,51,52,53]. The obtained temperature range in our film can basically fulfil the requirement. Furthermore, as more attention is paid to electronics technology, the requirement of reliability under high/low frequencies is highlighted. The room temperature frequency dependent *P-E* loops are displayed in Figure 3c. When the measured frequency rises from 500 Hz to 20 kHz, the changes of the *W*_rec_ and *η* values are only 9% and 2%, respectively, as shown in Figure 3d. Furthermore, the energy storage performance of the capacitor in long-term working conditions is also a key requirement for practical application. To evaluate its long-term charging–discharging stability, the fatigue endurance of BST/0.4BFO-0.6STO film is evaluated under 10 kHz at room temperature. The *P-E* loops of samples over 10^8^ charge–discharge cycles are exhibited in Figure 3e. It can be seen that there is no obvious change in the hysteresis loop. The corresponding *W*_rec_ and *η* present a negligible degradation of 6% and 2%, respectively, as shown in Figure 3f. The weak dependence of the energy storage performance on the temperature, frequency and fatigue cycles makes the BST/0.4BFO-0.6STO thin film more competent to work in different complex environments.

**Figure 4:**
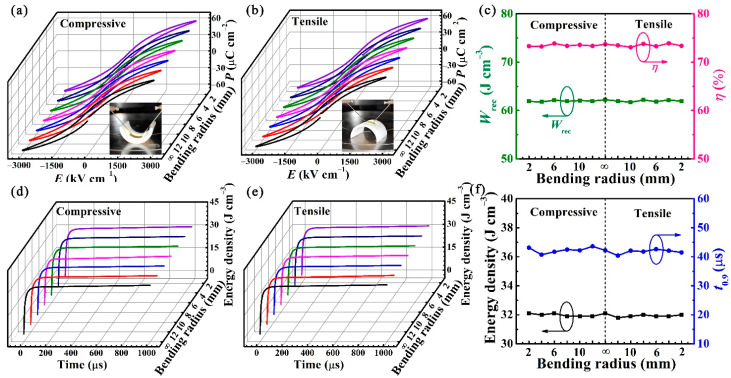
*P-E* loops measured at various (**a**) compressive radii and (**b**) tensile radii. The inset is the photographs of the BST/0.4BFO-0.6STO film under different bending states. (**c**) *W*_rec_ and *η* as functions of the bending radius. The energy discharge behaviors at various (**d**) compressive radii and (**e**) tensile radii. (**f**) Discharged energy density and discharge speed as functions of bending radius. (The lines in Figure 4a,b,d,e from black to purple represent the measurements of the bending radius of BST/0.4BFO-0.6STO film from ∞ to 2 mm, respectively.)

It is generally believed that the bending strain *S* can be calculated using the equation *S* = (*t*_f_ + *t*_s_)/2*r* [54,55], where *t*_f_ is the film thicknesses, *t*_s_ is the substrate thicknesses and *r* is the bending radius of the sample. The *t*_f_ and *t*_s_ for the BST/0.4BFO-0.6STO sample are ~350 nm and ~10 μm, respectively. Due to the limitations of stripping mica technology, the minimum bent radius of mica is 2 mm. In this curved state, the calculated *S* (~0.25%) is much less than the strain limit that the oxide film can withstand [56]. The mechanical stability of the BST/0.4BFO-0.6STO film is further evaluated under flex-in (compressive strain) and flex-out (tensile strain) modes at 2286 kV cm^−1^ and 10 kHz with different bending radii (from 12 mm to 2 mm), as depicted in Figure 4a,b. Then, home-made molds with different required bending radii are used to test mechanical stability. It can be seen that the *P-E* loops keep its slim feature without obvious deterioration regardless of what compressive strain or tensile strain it is under. As plotted in Figure 4c, when the bending radius decreases from 12 mm to 2 mm, the corresponding *W*_rec_ and *η* variations are both within 1%, indicating that the film possesses excellent bendability. The discharge energy density–time plots under various compressive and tensile radii are shown in Figure 4d,e. Obviously, all curves are very similar. Figure 4f shows the bending radius dependence of the discharged energy density and the discharge speed *t*_0.9_. The BST/0.4BFO-0.6STO film possesses a high discharged energy density (*W*_dis_) of ~32 J cm^−3^. Further, it can deliver the energy in ~40 μs without significant differences with the change of bending radius, exhibiting a fast charge–discharge rate and mechanical bending endurance.

**Figure 5:**
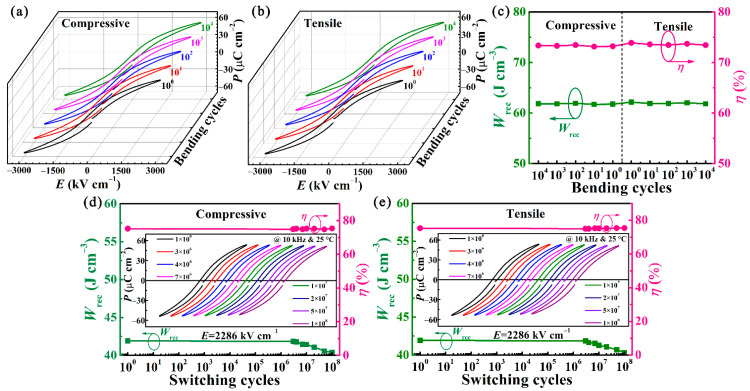
(**a**,**b**) *P*-*E* loops of BST/0.4BFO-0.6STO film under various bending cycles under the compressive and tensile states. (**c**) *W*_rec_ and *η* as functions of bending number. (**d**,**e**) *W*_rec_ and *η* as functions of switching cycle during 10^8^ fatigue cycles under compressive and tensile bending states with *r* = 4 mm. The insets are the corresponding *P-E* loops. (The lines in Figure 5a,b from black to green represent the measurements of the bending cycles of BST/0.4BFO-0.6STO film from 10^0^ to 10^4^, respectively.)

Figure 5a,b presents the *P-E* loops of the BST/0.4BFO-0.6STO sample in the flat and re-flatted after experiencing repeated bending at *r* = 4 mm. Over the course of 10^4^ cycles, nearly unchanged *P-E* hysteresis shapes are observed, guaranteeing high mechanical stability of the energy storage performances. As demonstrated in Figure 5c, the variations of the *W*_rec_ and *η* are negligible, further ascertaining its bending–endurance property. Finally, the influences of the ferroelectric fatigue endurance are investigated with *r* = 4 mm (Figure 5d,e). The energy storage performance is apparently undamaged even after 10^8^ switching cycles at a radius as small as of 4 mm.

**Figure 6:**
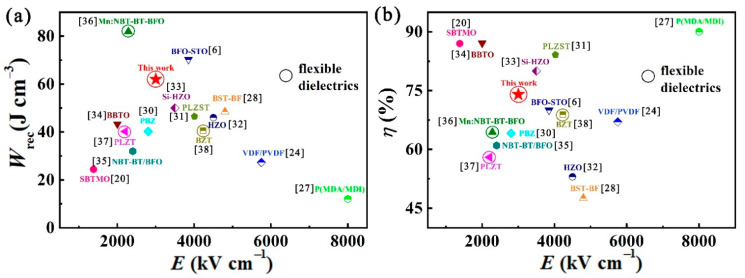
A comparison of the (**a**) *W*_rec_ and (**b**) *η* of the flexible BST/0.4BFO-0.6STO film capacitor reported in this study and a number of film capacitors reported previously.

Finally, the core parameters of *E*_b_, *W*_rec_ and *η* for energy storage properties are compared with some previously reported representative dielectrics (Figure 6). As depicted in Figure 6a, the BST/0.4BFO-0.6STO film exhibits relatively high *W*_rec_ of 62 J cm^−3^ at a moderate *E* of 3000 kV cm^−1^, which is much higher than HZO (46 J cm^−3^), BST-BF (48.5 J cm^−3^) and NBT-BT/BFO (31.96 J cm^−3^) [28,32,35], but slightly inferior to Mn: NBT-BT-BFO (81.9 J cm^−3^) and BFO-STO (70.3 J cm^−3^) [6,36]. In Figure 6b, it can be seen that the obtained *η* of 74% in this work is lower than the reported dielectrics on rigid substrate, such as SBTMO (87%), BBTO (87.1%) and PLZST (84%) [20,31,34] but reaches a relatively high level among all the currently reported bendable inorganic dielectric film capacitors. In view of the aforesaid observations, there is still much room for improvement of the *η* in the flexible film capacitors, and it needs further research.

## 4. Conclusions

In this work, a high *W*_rec_ of ~62 J cm^−3^ and an *η* of ~74% are achieved in the BST/0.4BFO-0.6STO film. The film shows superior thermal stability (from −50 to 200 °C), frequency reliability (from 500 Hz to 20 kHz) and fatigue endurance (10^8^ cycles). Most importantly, prominent mechanical stability can also be obtained. The energy storage behaviors show no obvious deterioration after undergoing different bending radii (from 12 to 2 mm), and even after 10^4^ bending cycles. All of these outstanding performances demonstrate that the designed flexible BST/0.4BFO-0.6STO thin film is expected to pave the way for its application in flexible energy storage electronic devices.

## Figures and Tables

**Table 1:** Comparison of energy storage performance of different types of materials.

Materials		Substrate	*P*_m_-*P*_r_ (μC/cm^2^)	*E* (kV·cm^−1^)	*W_rec_* (J·cm^−3^)	*η* (%)	*T* (°C)	Fatigue (Cycles)	Bending Test	Ref.
organic	P(VDF-TrFE-CTFE)		~10	4000	9		<150			[19]
	VDF/PVDF		~8	8200	27.3	67	<85			[24,25]
	P(VDF-CTFE)		9	5750	17					[26]
	P(MDA/MDI)		~3.2	8000	12	>90%	RT-180			[27]
inorganic	BST-BF	Pt/Ti/SiO_2_/Si	~30	4800	48.5	47.57	30–120			[28]
	SBTMO	Pt/Ti/SiO_2_/Si	34.3	1380	24.4	87	30–110			[20]
	BFO-STO	Nb:SrTiO_3_	~45	3850	70.3	70	−50–100	10^7^		[6]
	BZT/BZT	Nb:SrTiO_3_	~33	7500	83.9	78.4	−100–200	10^6^		[29]
	PBZ	Pt/TiO_x_/SiO_2_/Si	65	2801	40.18	64.1	−23–250			[30]
	PLZST	(La_0.7_Sr_0.3_)MnO_3_/Al_2_O_3_(0001)	55	4000	46.3	84	27–107	10^5^		[31]
	HZO	SiO_2_/Si	30	4500	46	53	25–175	10^9^		[32]
	Si-HZO	Si		3500	50	80	25–125	10^9^		[33]
	BBTO	Pt/Si	40	2000	43.3	87.1	20–140	10^8^		[34]
	NBT-BT/BFO	Pt/TiO_x_/SiO_2_/Si	43.19	2400	31.96	61	25–120			[35]
	Mn:NBT-BT-BFO	Pt/F-mica	97.8	2285	81.9	64.4	25–200	10^9^	r = 2 mm or 10^3^ at r = 4 mm	[36]
	PLZT	LaNiO_3_/F-Mica	~64	1998	40.2	58	30–180	10^7^	2 × 10^3^ at r = 4.5 mm	[37]
	BZT	Indium Tin Oxide (ITO)/F-mica	~25	4230	40.6	68.9	−120–150	10^6^	r = 4 mm or 10^3^ at r = 4 mm	[38]
	BST/0.4BFO-0.6STO	Pt/F-mica	56.79	3000	62	74	−50–200	10^8^	r = 2 mm or 10^4^ at r = 4 mm	This work

Poly(vinylidene fluoride-trifluoroethylene-chlorofluoroethylene) (P(VDF-TrFE-CTFE), vinylidene fluoride/Poly(vinylidene fluoride) (VDF/PVDF), Poly(vinylidene fluoride- chlorofluoroethylene) (P(VDF-CTFE), poly(diaminodiphenylmethane-diphenylmethane diisocyanate) (P(MDA/MDI), 0.1BiFeO_3_-0.9Bi_0.2_Sr_0.7_TiO_3_ (BST-BF), (Sr_0.85_Bi_0.1_)Ti_0.99_Mn_0.01_O_3_ (SBTMO), 0.4BiFeO_3_-0.6SrTiO_3_ (BFO-STO), BaZr_0.15_Ti_0.85_O_3_/BaZr_0.35_Ti_0.65_O_3_ (BZT/BZT), Pb_0.8_Ba_0.2_ZrO_3_ (PBZ), Pb_0.97_La_0.02_Zr_0.66_Sn_0.23_Ti_0.11_O_3_ (PLZST), Hf_0.3_Zr_0.7_O_2_ (HZO), Si-Hf_0.5_Zr_0.5_O_2_ (Si-HZO), BaBi_4_Ti_4_O_15_ (BBTO), 0.94(Bi_0.5_Na_0.5_)_0.94_TiO_3_-0.06BaTiO_3_/BiFeO_3_ (NBT-BT/BFO), 0.97(0.93Na_0.5_Bi_0.5_TiO_3_-0.07BaTiO_3_)-0.03BiFeO_3_ (Mn:NBT-BT-BFO), Pb_0.91_La_0.09_(Zr_0.65_Ti_0.35_)_0.9775_O_3_ (PLZT), Ba(Zr_0.35_Ti_0.65_)O_3_ (BZT), Ba_0.5_Sr_0.5_TiO_3_/0.4BiFeO_3_-0.6SrTiO_3_ (BST/0.4BFO-0.6STO).
